# Predictors of Surgical Margin Following Breast-Conserving Surgery: A Large Population-Based Cohort Study

**DOI:** 10.1245/s10434-016-5532-5

**Published:** 2016-09-02

**Authors:** Carolien H. M. van Deurzen

**Affiliations:** Department of Pathology, Erasmus MC Cancer Institute, Rotterdam, The Netherlands

## Abstract

**Background:**

The purpose of this retrospective, population-based, cohort study was to identify patient and tumor characteristics that are associated with a high risk of tumor-positive margins after breast-conserving surgery (BCS) to optimize preoperative counseling.

**Methods:**

All patients with invasive breast cancer (IBC) reported according to the synoptic reporting module in the Dutch Pathology Registry between 2009 and 2015 were included (*n* = 42.048 cases). Data extraction included age, type of surgery, several tumor characteristics, and resection margin status according to the Dutch indications for re-excision (free, focally positive, or more than focally positive). Univariate and multivariate tests were used to determine the association between clinicopathological features and margin status, restricted to patients with BCS.

**Results:**

Of 42,048 cases, a total of 25,315 cases (60.2 %) with IBC underwent BCS. Of these patients, 2578 patients (10.2 %) had focally positive resection margins and 1665 (6.6 %) had more than focally positive resection margins. By univariate analysis, the following features were significantly associated with involved margins: age < 60 years, multifocality, lobular subtype, tumor size >2 cm, intermediate- and high-grade, positive ER status, positive Her2 status, angio-invasion, and the presence/extent of a ductal carcinoma in situ (DCIS) component. In multivariate logistic regression, the variables with the strongest association with involved margins (OR > 2) were multifocality, lobular subtype, large tumor size, and the presence of DCIS.

**Conclusions:**

Several clinicopathologic features are associated with involved resection margins after BCS for IBC. Assessment of these features preoperatively could be used to optimize preoperative counseling.

Patients with early invasive breast cancer (IBC) who undergo breast-conserving surgery (BCS) followed by radiotherapy have a survival that is similar to patients who undergo a mastectomy.^1^,^2^ BCS provides better cosmesis compared with a mastectomy, but patients with BCS have a higher risk of involved resection margins, which is associated with an increased local recurrence risk.^3^,^4^ However, there is no international consensus regarding the definition of an optimal resection margin after BCS, resulting in substantial variation in surgical practice worldwide.^5^,^6^ Clearly, secondary re-excisions to attain wider margins result in increased costs, complications, discomfort, and have a negative effect on cosmesis.

Recently, the Society of Surgical Oncology (SSO) and the American Society for Radiation Oncology (ASTRO) published a multidisciplinary consensus guideline regarding surgical margins for patients with Stage I and II IBC undergoing BCS and whole-breast irradiation.^7^ According to this guideline, no ink on the tumor is regarded as the standard for an adequate margin. This is in line with the results of a meta-analysis that confirmed that local recurrences are reduced by negative resection margins.^6^ Increasing the distance required for the definition of a negative resection margin was weakly associated with a reduced local recurrence rate, although this effect was not significant after adjustment for covariates. In the Netherlands, however, the indications for a re-excision are even less stringent. According to the Dutch treatment guideline for breast cancer, margin status is defined as free (no ink on tumor), focally positive, or more than focally positive.^8^ Re-excision is indicated only for those patients with a more than focally positive resection margin. In contrast, patients with a focally positive resection margin do not undergo re-excision according to this guideline, because a radiation-boost is held to result in adequate local control and overall survival.^9^–^11^

Additional studies or consensus meetings could lead to a more uniform definition of optimal resection margin status and indication for re-excision, but regardless of the exact definitions, tumor-positive resection margins are regarded as a strong predictor for local recurrence. Therefore, preoperative prediction of the likelihood of positive resection margins could result in improved counseling regarding surgery and potentially a reduction in the number of re-excisions. Several studies reported on predictive factors for surgical margin status in BCS.^12^–^16^ However, the majority of these studies are relatively small, single-center studies, hampering the utility of the findings in daily practice. In the Netherlands, synoptic reporting of breast cancer is used, which offers a unique opportunity for a large, nationwide, retrospective cohort study. In this study, we report several clinical and pathologic factors that are associated with focal or more than focally positive resection margins after BCS, providing important information that can be used to optimize preoperative counseling.

## Patients and Methods

### Data Acquisition

In the Netherlands, all pathology reports are archived in the Dutch Pathology Registry (PALGA).^17^ Since 2009, synoptic reporting modules for reporting several common tumor types have been available, including breast cancer. In these modules, the parameters are captured in numerous variables instead of free text fields, which offers the opportunity to analyze all reports simultaneously.

### Patient and Tumor Characteristics

All consecutive patients with IBC reported according to the protocol-module in the Netherlands between January 1, 2009 and September 1, 2015 were included (*n* = 42,048 cases). We excluded patients with pure DCIS or patients with IBC after previous treatment (re-excisions after a previous irradical resection, neoadjuvant therapy). Patients with bilateral IBC were included as two cases. Where there was multifocality of tumor in one breast, the largest IBC was included for analysis of tumor characteristics. Resection margin status was assessed for all tumors in these cases.

Data extraction included age, type of surgery (BCS or mastectomy), tumor size, histological type (according to the WHO), grade (according to the modified Bloom and Richardson grading system), ER status, PR status, Her2 status, the presence, and extent of DCIS. ER and PR status were defined as positive where more than 10 % of the cancer cells showed nuclear staining, irrespective of density, according to the Dutch Guideline for breast cancer treatment.^8^ Her2 status was scored according to international guidelines.^18^

The overall resection margin status was reported as free, focally irradical, or more than focally irradical, according to the Dutch Guideline for Breast Cancer Treatment. A free resection margin is defined as no tumor reaching the ink. Focally irradical is defined as tumor (either invasive or DCIS) reaching in the ink in a small area (≤4 mm). When the tumor (either invasive or DCIS) reaches the ink in a larger area or multiple smaller areas, it is defined as more than focally irradical.

### Statistical Analysis

Univariate and multivariate logistic regression analyses were performed to test for associations between clinicopathologic features and positive resection margins. Variables that were significant in the univariate analysis were included in a multivariate model, excluding those variables that cannot reliably be assessed preoperatively (presence and extent of DCIS outside the invasive component). Analyses were restricted to patients undergoing BCS. Because there is no international consensus regarding indications for re-excision, we preformed these analyses according to two different methods. First, we compared those patients with a free or focally positive margin to patients with more than focally positive margins. Second, because several countries use the definition of “no ink on tumor” to define resection margin status, we compared patients with free margins to those with involved margins (either focally or more than focally).

Two sided *p* values < 0.05 were considered significant. All analyses were performed with SAS Enterprise Guide 7.1.

## Results

### Patients

Overall, we included 42,048 cases of IBC reported between January 1, 2009 and September 1, 2015. The median age of our patient cohort was 62 years (range 18–100). The majority of patients (25,315/42,048; 60.2 %) underwent BCS. Table 1 provides an overview of clinicopathologic data of all patients.

**Table 1:** Baseline characteristics of all patients with IBC (*n* = 42,048)

Characteristic	Total number of tumors (*n* = 42.048)
Age at diagnosis	Mean 61.2
	Median 62 (range 18–100)
Type of surgery, no (%)	
Breast-conserving surgery	25.315 (60.2)
Mastectomy	16.733 (39.8)
Tumor type, no (%)	
Ductal	33.795 (80.4)
Lobular	5.250 (12.5)
Other	3.003 (7.1)
Multifocality	
Yes	3.051 (8.9)
No	31.296 (91.1)
Missing	7.701
Tumor size, no (%)	
≤2 cm	27.737 (66.0 )
>2–≤5 cm	12.656 (30.1)
>5 cm	1.651 (3.9)
Missing	4
Tumor grade, no (%)	
1	9.600 (27.0)
2	16.563 (46.6)
3	9.398 (26.4)
Missing	6.487
Estrogen receptor status, no (%)	
Positive	34.393 (85.4)
Negative	5.861 (14.6)
Missing	1794
Progesterone receptor status, no (%)	
Positive	27.697 (69.0)
Negative	12.462 (31.0)
Missing	1.889
Her2 status, no (%)	
Positive	4.224 (11.3)
Negative	33.134 (88.7)
Missing	4.690
Angio-invasion, no (%)	
Yes	4.118 (14.0)
No	25.304 (86.0)
Missing	12.626
Presence of DCIS, no (%)	
Yes	18.140 (43.1)
No	23.908 (56.9)
DCIS restricted to invasive component, no (%)	
Yes	5.882 (54.7)
No	4.878 (45.3)
Missing	7.380
Diameter of DCIS, no (%)	
≤2 cm	7.982 (67.4)
>2–≤5 cm	2.968 (25.0)
>5 cm	896 (7.6)
Missing	6.294

### Resection Margin Status

Resection margin status was reported for the majority of patients undergoing BCS (25,311/25,315 patients). Table 2 presents the association between several clinicopathological variables and a more than focally involved resection margin. Overall, 1665 patients (6.6 %) had more than focally positive resection margins. Briefly, the following variables were associated with increased risk of more than focally positive resection margins in univariate analysis: age <60 years, multifocality, lobular subtype, large tumor size (>2 cm), intermediate- and high-grade, positive ER status, positive Her2 status, angio-invasion, and the presence/extent of a DCIS component. The following variables were significantly associated with more than focally involved margins in multivariate analysis: age <50 years, multifocality, lobular subtype, size >2 cm, angio-invasion, and the presence of a DCIS component. The strongest effect (OR > 2) was seen for multifocality, lobular subtype, size >2 cm, and the presence of DCIS.

**Table 2:** Patient and tumor characteristics of all patients undergoing BCS with available resection margin status (*n* = 25,311)

	Total (*n* = 25.311)	Margin free or focally irradical	Margin more than focally irradical	OR, univariate analysis (95 % CI)	OR, multivariate analysis (95 % CI)
Age at diagnosis, no (%)					
<50	4390	4010 (91.34)	380 (8.66)	1.53 (1.35–1.74)	1.22 (1.00–1.49)
50–59	7085	6607 (93.25)	478 (6.75)	1.17 (1.04–1.31)	1.01 (0.85–1.20)
≥60	13836	13029 (94.17)	807 (5.83)	1.0 (ref)	1.0 (ref)
Multifocality, no (%)					
Yes	773	637 (82.41)	136 (17.59)	3.25 (2.68–3.95)	2.82 (2.14–3.71)
No	19911	18685 (93.84)	1226 (6.16)	1.0 (ref)	1.0 (ref)
Missing	4627				
Tumor type, no(%)					
Ductal	21143	19889 (94.07)	1254 (5.93)	1.0 (ref)	1.0 (ref)
Lobular	2344	2039 (86.99)	305 (13.01)	2.37 (2.08–2.71)	3.12 (2.48–3.92)
Other	1824	1718 (94.19)	106 (5.81)	0.98 (0.80–1.20)	1.18 (0.88–1.58)
Tumor size, no (%)					
≤2 cm	19816	18807 (94.91)	1009 (5.09)	1.0 (ref)	1.0 (ref)
>2–≤5 cm	5351	4754 (88.84)	597 (11.16)	2.34 (2.11–2.60)	2.01 (1.71–2.37)
>5 cm	144	85 (59.03)	59 (40.97)	12.95 (9.24–18.16)	12.30 (7.12–21.26)
Tumor grade, no (%)					
1	6720	6401 (95.25)	319 (4.75)	1.0 (ref)	1.0 (ref)
2	9747	9032 (92.66)	715 (7.34)	1.59 (1.39–1.82)	1.19 (0.99–1.43)
3	5108	4768 (93.34)	340 (6.66)	1.43 (1.22–1.68)	1.03 (0.81–1.31)
Missing	3736				
Estrogen receptor status, no (%)					
Positive (≥10%)	21182	19739 (93.19)	1443 (6.81)	1.0 (ref)	1.0 (ref)
Negative (<10%)	3130	2962 (94.63)	168 (5.37)	0.78 (0.66–0.92)	0.94 (0.72–1.21)
Missing	999				
Progesterone receptor status, no (%)					
Positive (≥10%)	16769	15654 (93.35)	1115 (6.65)	1.0 (ref)	
Negative (<10%)	7518	7024 (93.43)	494 (6.57)	0.99 (0.89–1.10)	
Missing	1024				
Her2 status, no (%)					
Positive	2152	1972 (91.64)	180 (8.36)	1.0 (ref)	1.0 (ref)
Negative	20416	19094 (93.52)	1322 (6.48)	0.76 (0.65–0.89)	0.82 (0.64–1.05)
Missing	2743				
Angio-invasion, no (%)					
Yes	1784	1581 (88.62)	203 (11.38)	2.02 (1.72–2.37)	1.63 (1.33–2.01)
No	16105	15142 (94.02)	963(5.98)	1.0 (ref)	1.0 (ref)
Missing	7422				
Presence of DCIS, no (%)					
Yes	11233	10205 (90.85)	1028 (9.15)	2.13 (1.91–2.35)	2.71 (2.29–3.22)
No	14078	13441 (95.48)	637 (4.52)	1.0 (ref)	1.0 (ref)
DCIS outside invasive component, no (%)					
Yes	3694	3251 (88.01)	443 (11.99)	2.42 (2.01–2.92)	
No	3037	2875 (94.67)	162 (5.33)	1.0 (ref)	
Missing	4502				
Diameter of DCIS, no (%)					
≤2 cm	5594	5262 (94.07)	332 (5.93)	1.0 (ref)	
>2–≤5 cm	1526	1215 (79.62)	311 (20.38)	4.06 (3.43–4.79)	
>5 cm	196	125 (63.78)	71 (36.22)	9.00 (6.59–12.30)	
Missing	3917				

We also performed this analysis by comparing free margin to involved margin (either focally or more than focally). Table 3 provides an overview of clinicopathological variables related to involved margins. Overall, 4243 patients (16.8 %) had either focally or more than focally positive resection margins. In general, univariate results were similar to those presented in Table 2; exactly the same variables that were associated with more than focally involved margins were found when both focally or more than focally involved margins were analyzed together, although odd ratios and confidence intervals were slightly different. Results of multivariate analysis were also comparable to the results reported in Table 2, although small differences were seen. In multivariate analyses, the following variables were significantly associated with focally or more than focally involved margins: age <50 years, multifocality, lobular subtype, size >2 cm, grade 2, positive ER status, positive Her2 status, angio-invasion, and the presence of a DCIS component. The strongest effect (OR > 2) was seen for multifocality, lobular subtype, size >5 cm, and the presence of DCIS.

**Table 3:** Patient and tumor characteristics of all patients undergoing BCS with available resection margin status (*n* = 25,311)

	Total (*n* = 25.311)	Margin free (*n* = 23.646)	Margin focally or more than focally irradical (*n* = 1665)	OR, univariate analysis (95 % CI)	OR, multivariate analysis (95 % CI)
Age at diagnosis, no (%)					
<50	4390	3517 (80.11)	873 (19.89)	1.33 (1.22–1.45)	1.15 (1.00–1.31)
50–59	7085	5889 (83.12)	1196 (16.88)	1.09 (1.01–1.18)	1.05 (0.94–1.18)
≥60	13836	11662 (84.29)	2174 (15.71)	1.0 (ref)	
Multifocality, no (%)					
Yes	773	512 (66.24)	261 (33.76)	2.66 (2.28–3.11)	2.59 (2.09–3.20)
No	19911	16713 (83.94)	3198 (16.06)	1.0 (ref)	1.0 (ref)
Missing	4627				
Tumor type, no(%)					
Ductal	21143	17777 (84.08)	3366 (15.92)	1.0 (ref)	1.0 (ref)
Lobular	2344	1735 (74.02)	609 (25.98)	1.85 (1.68–2.05)	2.79 (2.38–3.28)
Other	1824	1556 (85.31)	268 (14.69)	0.91 (0.80–1.04)	1.13 (0.93–1.36)
Tumor size, no (%)					
≤2 cm	19816	16904 (85.30)	2912 (14.70)	1.0 (ref)	1.0 (ref)
>2–≤5 cm	5351	4095 (76.53)	1256 (23.47)	1.78 (1.65–1.92)	1.59 (1.42–1.79)
>5 cm	144	69 (47.92)	75 (52.08)	6.31 (4.54–8.77)	5.94 (3.49–10.11)
Missing					
Tumor grade, no (%)					
1	6720	5808 (86.43)	912 (13.57)	1.0 (ref)	1.0 (ref)
2	9747	8007 (82.15)	1740 (17.85)	1.38 (1.27–1.51)	1.19 (1.06–1.34)
3	5108	4231 (82.83)	877 (17.17)	1.32 (1.19–1.46)	1.14 (0.97–1.33)
Missing	3736				
Estrogen receptor status, no (%)					
Positive (≥10 %)	21182	17522 (82.72)	3660 (17.28)	1.0 (ref)	1.0 (ref)
Negative (<10 %)	3130	2685 (85.78)	445 (14.22)	0.79 (0.71–0.88)	0.81 (0.69–0.97)
Missing	999				
Progesterone receptor status, no (%)					
Positive (≥10 %)	16769	13921 (83.02)	2848 (16.98)	1.0 (ref)	
Negative (<10 %)	7518	6267 (83.36)	1251 (16.64)	0.98 (0.91–1.05)	
Missing	1024				
Her2 status, no (%)					
Positive	2152	1696 (78.81)	456 (21.19)	1.0 (ref)	1.0 (ref)
Negative	20416	17062 (83.57)	3354 (16.43)	0.73 (0.66–0.82)	0.83 (0.70–0.97)
Missing	2743				
Angio-invasion, no (%)					
Yes	1784	1324 (74.22)	460 (25.78)	1.89 (1.68–2.12)	1.56 (1.35–1.80)
No	16105	13600 (84.45)	2505 (15.55)	1.0 (ref)	1.0 (ref)
Missing	7422				
Presence of DCIS, no (%)					
Yes	11233	8655 (77.05)	2578 (22.95)	2.22 (2.08–2.38)	2.76 (2.48–3.08)
No	14078	12413 (88.17)	1665 (11.83)	1.0 (ref)	1.0 (ref)
Missing					
DCIS restricted to invasive component, no (%)					
Yes	4120	2884 (70.00)	1236 (30.00)	2.71 (2.41–3.05)	
No	3325	2871 (86.35)	454 (13.65)	1.0 (ref)	
Missing	3788				
Diameter of DCIS, no (%)					
≤2 cm	5594	4551 (81.36)	1043 (18.64)	1.0 (ref)	
>2–≤5 cm	1526	889 (58.26)	637 (41.74)	3.12 (2.77–3.53)	
>5 cm	196	78 (39.80)	118 (60.20)	6.60 (4.92–8.85)	
Missing	3917				

## Discussion

The majority of patients with IBC are treated with BCS, followed by irradiation. One of the challenges regarding BCS is to attain tumor-free resection margins to decrease the number of second operations and improve local control. In this large population-based cohort study, we reported several patient and tumor characteristics that are significantly associated with an increased rate of tumor-positive resection margins (irrespective of the definition of irradicality), in particular multifocality, lobular subtype, tumor size >2 cm, and the presence of a DCIS component. Because these features can be assessed preoperatively by imaging and needle biopsy, this provides the opportunity to improve preoperative counseling regarding optimal surgery. This could reduce the number of re-excisions in those patients with a substantial risk of involved margins (e.g., those with a large lobular carcinoma) by adjustment of local therapy.

Histologic grade and receptor status were only significantly associated with irradicality in multivariate analyses by comparing patients with free margins to patients with any involved margins. Receptor status can reliably be determined on a biopsy specimen.^19^,^20^ Histologic grade, however, may be underestimated, which may not assist in preoperative counseling.^21^ There is an increased risk of irradicality when ER or Her2 receptors are positive. PR positivity showed the same trend; however, it was not statistically significant.

Based on a biopsy, the presence of DCIS and angio-invasion also can be assessed, and, if present, provide additional information regarding the risk of irradicality. Obviously, the biopsy only represents a part of the tumor, so if these factors are absent on the biopsy, it could still be present in the excision specimen, limiting the value of these factors preoperatively. Assessment of the extent of DCIS also is to some extent possible based on a preoperative needle biopsy and imaging. However, this is not entirely reliable, because a biopsy usually represents the central invasive component (whereas DCIS can be more extensive surrounding the invasive component) and imaging modalities are suboptimal for preoperative size estimation of the DCIS component. Breast imaging by preoperative MRI is the most sensitive method for estimating the extent of the DCIS component, mainly in the case of high-grade DCIS, but MRI is not routinely preformed for all patients undergoing BCS.^22^,^23^

Our findings are consistent with the literature. However, the strength of our study is that it represents a nationwide, consecutive, large series of patients with IBC, resulting in the largest series published on this subject. One limitation is that the Dutch distinction between focal and more than focal tumor-positive resection margins is not applied in most other European and North American countries. We adjusted for this by analyzing our data according to both the Dutch guideline and the SSO/ASTRO guideline, which defines an adequate margin of IBC as the absence of tumor reaching the ink. A second limitation is the lack of clinical follow-up regarding local recurrence and survival, due to the fact that synoptic reporting only began in 2009. Finally, a substantial proportion (>10 %) of data were missing for the following variables: multifocality, grade, Her2 status, angio-invasion, presence, and extent of DCIS outside the invasive component.

## Conclusions

In this large study, we identified several clinical and pathological factors that are significantly associated with involved resections margins after BCS for IBC. Because the majority of these features are assessed preoperatively, this provides the opportunity for an optimal preoperative risk prediction and possibly adjustment of surgical method.
